# A Herbal Formula, CGXII, Exerts Antihepatofibrotic Effect in Dimethylnitrosamine-Induced SD Rat Model

**DOI:** 10.1155/2016/5093718

**Published:** 2016-05-29

**Authors:** Hyo-Seon Kim, Hyeong-Geug Kim, Hye-Won Lee, Sung-Bae Lee, Jin-Seok Lee, Hwi-Jin Im, Won-Yong Kim, Dong-Soo Lee, Chang-Gue Son

**Affiliations:** ^1^Liver and Immunology Research Center, Daejeon Oriental Hospital, Oriental Medical College, Daejeon University, 176-9 Daeheung-ro, Jung-gu, Daejeon 34929, Republic of Korea; ^2^TKM-Based Herbal Drug Research Group, Korea Institute of Oriental Medicine, Daejeon 34054, Republic of Korea; ^3^Department of Internal Medicine, Daejeon St. Mary's Hospital, The Catholic University of Korea, 64 Daeheung-ro, Jung-gu, Daejeon 34943, Republic of Korea

## Abstract

We aimed to evaluate the antihepatofibrotic effects of CGXII, an aqueous extract which is composed of* A. iwayomogi*,* A. xanthioides*, and* S. miltiorrhiza*, against dimethylnitrosamine- (DMN-) induced hepatofibrosis. Male Sprague Dawley rats were intraperitoneally injected with 10 mg/kg of DMN for 4 weeks (three consecutive days weekly). Rats were orally given distilled water, CGXII (50 or 100 mg/kg), or dimethyl dimethoxy biphenyl dicarboxylate (50 mg/kg) daily. DMN injection caused substantial alteration of total body weight and liver and spleen mass, whereas they were notably normalized by CGXII. CGXII treatment also markedly attenuated the elevation of serum aspartate aminotransferase and alanine aminotransferase levels, hepatic lipid peroxidation, and protein carbonyl contents. Collagen accumulation in hepatic tissue evidenced by histopathological analysis and quantitative assessment of hepatic hydroxyproline was ameliorated by CGXII. Immunohistochemistry analysis revealed decreased *α*-smooth muscle actin supporting the antihepatofibrotic effect of CGXII. The profibrogenic cytokines transforming growth factor-*β*, platelet-derived growth factor-*β*, and connective tissue growth factor were increased by DMN injection. Administration of CGXII normalized the protein and gene expression levels of these cytokines. Our findings suggest that CGXII lowers the levels of profibrogenic cytokines and thereby exerts antifibrotic effects.

## 1. Introduction

Hepatic fibrosis is the critical step in the progression of chronic liver disease because it determines the clinical outcome to recovery or progression to liver cirrhosis or hepatocellular carcinoma [1]. Hepatic fibrosis is a common wound healing response to chronic liver injuries, including hepatitis viral infections, toxic agent invasions, and alcohol abuse [2–4]. In hepatic fibrosis, hepatic stellate cells (HSCs) can form excessive extracellular matrix (ECM) that includes collagen and *α*-SMA [5]. Additionally, continuous HSC activation leads to myofibroblast transition via the release of pro-fibrogenic cytokines including transforming growth factor (TGF)-*β*, platelet-derived growth factor (PDGF)-*β*, and connective tissue growth factor (CTGF) [6, 7]. Thus, the HSC activations mainly affect the therapeutics response to liver fibrosis.

Despite many efforts to elucidate the pathophysiology of hepatofibrosis, hitherto no effective treatment or therapeutic drug has been established [8, 9]. Thus, a realistic strategy would be to prevent the underlying disease and inhibit fibrotic progression [10]. Additionally, herbal medicine has been expected to play a role in antihepatofibrosis treatment to improve quality of life (QOL) of patients with chronic liver disorders [11, 12].

To treat hepatofibrosis, various herbal medicines have been prescribed for thousands of years in clinical practice. The* Artemisia iwayomogi *Kitamura,* Amomum xanthioides *Wallich, and* Salvia miltiorrhiza *Bunge have been most frequently used to treat especially the chronic liver diseases, due to their efficacies. According to the Traditional Oriental Medicine (TOM) theory, hepatofibrosis is generally attributed to impairment of two critical liver functions: metabolic activities and blood homeostasis [13, 14]. The abovementioned three herbal plants have demonstrated pharmacological efficacies using the animal-based pathophysiological conditions of “*dampness and Phlegm*” [15], “*stagnation of vital energy*” [16], and “*blood stasis*” [17], respectively. Based on data from clinical practice and animal-based studies, the CGXII mixture used in these experiments was a water extract mixture of* A. iwayomogi*,* A. xanthioides*, and* S. miltiorrhiza* prepared at an equal ratio [18, 19].

In order to support the clinical relevance of CGXII, we adapted a DMN-induced hepatofibrosis rat model and used dimethyl biphenyl dicarboxylate (DDB) as a reference compound, which is well known to be potent antihepatotherapeutics [20, 21]. We herein evaluated the antihepatofibrotic properties of CGXII and explored its underlying mechanisms responsible for the action of CGXII.

## 2. Materials and Methods

### 2.1. Reagents and Chemicals

Dimethylnitrosamine (N-nitrosodimethylamine or DMN), 1,1,3,3-tetraethoxypropane (TEP), chloramine-T,* p*-dimethylaminobenzaldehyde, hydroxyproline, potassium chloride (KCl), and trichloroacetic acid (TCA) were purchased from Sigma (St. Louis, MO, United States); Thiobarbituric acid (TBA) was purchased from Lancaster Co. (Lancashire, United Kingdom). Hydrochloric acid and phosphoric acid were from Kanto Chemical Co., Inc. (Tokyo, Japan);* n*-butanol was purchased from J. T. Baker (Center Valley, PA, United States); Mayer's haematoxylin and isopropanol were obtained from Wako Pure Chemical Industries (Osaka, Japan); Goat anti-human connective tissue growth factor (CTGF) antibody, CTFG standard solution, rabbit anti-human CTGF antibody, and anti-rabbit immunoglobulin G horseradish peroxidase conjugate were purchased from Santa Cruz Biotechnology (Santa Cruz, CA, United States), and DDB was purchased from Pharma King Co., Ltd. (Gyeonggi-do, Republic of Korea).

### 2.2. Preparation of CGXII and Its Fingerprinting Analysis

*A. iwayomogi*,* A. xanthioides*, and* S*.* miltiorrhiza *were obtained from the Jeong-Seong Oriental Medicine Company (Daejeon, Republic of Korea). Briefly, 100 g of each of the three fully dried herbs was mixed and boiled separately in distilled water for 90 minutes and concentrated nonstop for 120 minutes. After filtration and lyophilization, the extracts were stored at −70°C until use. The final yield of water extraction was 9.58%.

The reference standard stock solutions of quercitrin, quercetin-dehydrate, rosmarinic acid, salvianolic acid B, scopoletin, and tanshinone IIA (each component was dissolved at 250 *μ*g/mL in 90% methanol) were prepared in methanol and stored at −4°C. The standard solutions were prepared using six concentrations of diluted solutions. All calibration curves were made by assessing the peak areas in the range of 2.5–500 *μ*g/mL. The linearity of the peak area (*y*) versus concentration (*x*, *μ*g/mL) curve for each component was used to calculate the contents of the main components in CGXII.

A quantitative analysis was performed under the simultaneous conditions using an 1100 series high-performance liquid chromatography (HPLC, Agilent Technologies, Santa Clara, CA) equipped with an autosampler (G11313A), column oven (GA1316A), binary pump (G1312), diode-array-detector (G1315B), and degasser (G1379A). The analytical column with a Kinetex C18 (4.6 × 250 nm, particle size 5 *μ*m, Phenomenex, Torrance, CA) was kept at 30°C during the experiment. The data were acquired and processed by ChemStation software (Agilent Technologies, USA). The mobile phase conditions contained 10% acetonitrile in distilled water (DW) with 0.05% formic acid (A) and 90% acetonitrile in water (B). The gradient flow was as follows: 0–30 min, 0%–20% B; 30–50 min, 20%–75% B; 50–60 min, 75%–100% B. The analysis was operated at a flow rate of 1.0 mL/min and detected at 280 nm. The injection volume was 10 *μ*L.

### 2.3. Animals and Experimental Schedule

The design and performance of the experiments were approved by the Institutional Animal Care and Use Committee of Daejeon University (DJUARB2015-006) and conducted in accordance with the Policy on the Humane Care and Use of Laboratory Animals, as adopted and promulgated by the US National Institute of Health (NIH).

A total of 30 heads of specific pathogen-free SD rats (6-week-old, 190–210 g) were purchased from Daehan-Biolink (Chungbuk, Republic of Korea) and housed in a controlled temperature room at 22 ± 2°C, 55%  ±  10% relative humidity, 12-hour light/dark cycles, and freely fed commercial pellets (Daehan-Biolink, Chungbuk, Republic of Korea) and tap water* ad libitum* for 7 days. After acclimation, rats were randomly divided into the following 5 groups (*n* = 6 for each group): normal (DW without DMN injection), DMN (DW with DMN injection), CGXII 50 or 100 (CGXII 50 or 100 mg/kg with DMN injection), and DDB 50 (DDB 50 mg/kg with DMN injection) groups. All animals were orally given DW, CGXII (50, or 100 mg/kg), or DDB (50 mg/kg) by gastric gavage once daily for 4 weeks. The DMN was intraperitoneally injected on three consecutive days per week for 4 weeks (10 mg/kg, dissolved in neutral saline), except for the normal group. The normal group was intraperitoneally injected with neutral saline. The body weights were recorded twice weekly during the experiment. On the final experimental day after 12 hours of fasting, all of the rats were weighed and sacrificed under ether anesthesia. Whole blood was isolated from the abdominal aorta using syringes for biochemical analyses. The livers and spleens were removed, immediately weighed, and photographed. Liver tissues were either fixed in 10% formalin solution for histopathological examination or RNA later solution or stored at −70°C for gene expression analysis and biochemical analysis, respectively.

### 2.4. Complete Blood Count and Serum Biochemical Analysis

Blood was collected from the abdominal aorta on the final day of experiment. After centrifuging at 3000 ×g for 15 min, the serum was separated and stored at −70°C. The serum levels of total bilirubin, aspartate transaminase (AST), and alanine transaminase (ALT) were determined using an Auto Chemistry Analyzer (AU400, Olympus, Tokyo, Japan).

### 2.5. Histomorphological Analysis and Immunohistochemical Staining

For the histomorphological evaluations, a portion of liver tissue was fixed in 10% formalin solution and embedded in paraffin. The paraffin-embedded liver was sectioned (5 *μ*m thickness), deparaffinized, hydrated, and stained for hematoxylin & eosin (H&E) staining. Masson's trichrome staining was performed to distinguish cells from surrounding connective tissue.

For immunohistochemical staining of *α*-smooth muscle actin (*α*-SMA), liver tissue sections were deparaffinized, hydrated, and heated in antibody specific retrieval buffer (1 mM EDTA in 0.05% Tween 20) at 100°C for 5 min and then treated with goat serum for 30 min. The slides were then incubated overnight with an anti-*α*-SMA mouse monoclonal antibody (1 : 250, Abcam, Cambridge, UK) and incubated overnight. After washing with tap water, Histofine (Nichirei Biosciences, Tokyo, Japan) was added using TMB as a substrate. The slides were examined under an optical microscope (100x magnifications, Leica Microsystems, Wetzlar, Germany).

These histopathologic changes for inflammation were scored on a scale of 0 to 3, specifically 0 for normal state (<5% pathological changes), 1 for mild (<10%), 2 for moderate (15%–20%), and 3 for severe (>20%) [22]. A METAVIR fibrosis score from 0 to 4 was used to differentiate the levels of liver fibrosis. Briefly, stage 0 refers to no scarring, stage 1 to minimal scarring, stage 2 to scarring in other areas containing blood vessels than the liver, stage 3 to bridging fibrosis spread to other fibrotic areas, and stage 4 to advanced scarring of the liver or cirrhosis [23]. The percentage areas of positive *α*-SMA staining cells were analyzed by the image analysis program, ImageJ 1.46 software (Rasband, Bethesda, MD, USA).

### 2.6. Determination of Hydroxyproline, Lipid Peroxidation, and Protein Carbonyl in Liver Tissues

Hydroxyproline assays were performed as previously described with a slight modification [24]. Briefly, liver tissue (0.2 g) stored at −70°C was homogenized in 2 mL of 6 N HCl and incubated overnight at 110°C. After filtering the acid hydrolysates using filtering paper (Toyo Roshi Kaisha, Tokyo, Japan), 50 *μ*L of samples and standards was incubated to completely evaporate the HCl. Then 50 *μ*L of methanol and 1.2 mL of 50% of isopropanol were added after incubation, and 200 *μ*L of chloramine-T solution was sequentially added to the samples. After further incubation at room temperature for 10 min, 1.3 mL of Ehrlich's solution was added to the mixture which was incubated at 50°C for 90 min. At the end of the incubation, the absorbance of the reaction mixtures was determined at 558 nm. A standard curve was constructed using 0–1.0 mg/50 *μ*L of hydroxyproline solution.

Lipid peroxidation levels were evaluated by the thiobarbituric acid reactive substances (TBARS) method, as described previously [25]. Briefly, liver tissue (200 mg) was homogenized in ice-cold KCl (1.15%), and the homogenate was mixed with 1% H_3_PO_4_ and 0.67% TBA solution. The mixture was heated for 45 min at 100°C, *n*-butanol was added, and the solution was then mixed and centrifuged at 3000 ×g for 15 min. The absorbance of the supernatant was measured at 535 and 520 nm and compared to a standard value (freshly prepared TEP solution).

Hepatic protein carbonyl content was determined by detecting protein oxidation using the DNPH reaction, according to the previously described method [26]. Briefly, the liver tissue homogenate was prepared with cold phosphate buffer (50 mM, pH 6.7, containing 1 mM EDTA), and 200 *μ*L of the homogenate was mixed with 800 *μ*L of DNPH (10 mM dissolved in 2.5 M HCl). After incubation in the dark at room temperature for 1 h with vortexing every 15 min followed by the sequential addition of 1 mL of 20% TCA and 10% TCA and incubation for 5 min at 4°C, a pellet was obtained by centrifugation at 10,000 ×g for 10 min at 4°C. After resuspension in 1 mL of an ethanol/ethyl acetate mixture (1 : 1, v/v) and centrifugation, the protein pellets were re-suspended in 500 *μ*L of guanidine hydrochloride by vortexing and centrifugation at 10,000 ×g for 10 min at 4°C. Then, 220 *μ*L of the supernatants were transferred to a 96-well plate. The absorbance was measured at 370 nm with a spectrophotometer (Molecular Device Corp).

### 2.7. Determination of Profibrogenic Cytokines and Tissue Inhibitor of Metalloproteinase-1 (TIMP-1)

Transforming growth factor-beta1 (TGF-*β*1), platelet-derived growth factor-BB (PDGF-BB), and TIMP-1 levels in the liver tissues were measured using commercial ELISA kits (R&D Systems, Minneapolis, MN). We also manually measured the level of CTGF in hepatic tissues. Briefly, after coating 96-well plates with goat anti-human CTGF antibody, the plates were incubated with blocking buffer (10 mM PBS with sodium azide and 1% bovine albumin) for 1 h at room temperature. Then, 100 *μ*L of diluted homogenate samples and CTFG standard solution were added to the plates, followed by incubation for 1 h at room temperature. After binding of rabbit anti-human CTGF (100 *μ*L, 2 *μ*g/mL), 50 *μ*L of anti-rabbit IgG-HRP was added to each well, and incubation was continued for 1 h. To each well, 100 *μ*L of substrate solution was added and incubated for 20 min, followed by the addition of 50 *μ*L stop solution. The absorbance at 405 nm was read within 15 min. All antibodies used for the measurement of CTGF were purchased from Santa Cruz Biotechnology.

### 2.8. Real-Time PCR for Analyzing Gene Expression in Liver Tissues

Total RNA was extracted from liver tissue samples using the reagent Trizol (Molecular Research Center, Cincinnati, OH, USA). cDNA was synthesized from total RNA (2 *μ*g) in a 20 *μ*L reaction using the High-Capacity cDNA reverse transcription kit (Ambion, Austin, TX, USA). Real-time PCR was performed using SYBR Green PCR Master Mix (Applied Biosystems, Foster City, CA, USA) with PCR amplification performed in accordance with a standard protocol using the IQ5 PCR Thermal Cycler (Bio-Rad, Hercules, CA, USA). The primer sequences used were the following (shown 5′ → 3′): for *β*-actin, AGG CCA ACC GTG AAA AGA TG and CCA GAG GCA TAC AGG GAC AAC; for TGF-*β*1, AGC AGG AAG GGT CGG TTC AT and AGG AGA CGG AAT ACA GGG CTT T; for PDGF-BB, TGT GCT CGG GTC ATG TTC AA and ACC ACT CCA TCC GCT CCT TT; for CTGF, CAG TTG GCT CGC ATC ATA GTT G and GTG TGT GAT GAG CCC AAG GA; for collagen type 1 alpha (Col 1a1), GAT CCT GCC GAT GTC GCT AT and TGT AGG CTA CGC TGT TCT TGC A; for tissue inhibitor of metalloproteinase-1 (TIMP-1), ATG GAG AGC CTC TGT GGA TAT GTC and AGG CAG TGA TGT GCA AAT TTC C; for matrix metalloproteinase-2 (MMP-2), TGT GGC AGC CCA TGA GTT C and TCG GAA GTT CTT GGT GTA GGT GTA; for bone morphogenetic proteins and activating membrane-bound inhibitor (BAMBI), TTA TGT TGG CCT TGC GAA TG and TGG TGT CCA TGG AAG CTG TAG T; for Smad 7, TGC AAC CCC CAT CAC CTT AG and GAC AGT CTG CAG TTG GTT TGA GA. The final results are expressed as normalized fold values relative to the normal group.

### 2.9. Statistical Analysis

All data are expressed as the mean ± standard deviation (SD). Statistically significant differences between the groups were analyzed by one-way analysis of variance (ANOVA) followed by* post hoc* multiple comparison Fisher's least-significant difference (LSD) test using IBM SPSS version 20.0 (SPSS Inc. Chicago, IL, USA). Differences with *p* < 0.05, *p* < 0.01, or *p* < 0.001 were considered statistically significant.

## 3. Results

### 3.1. Fingerprinting Analysis of CGXII

**Figure 1:**
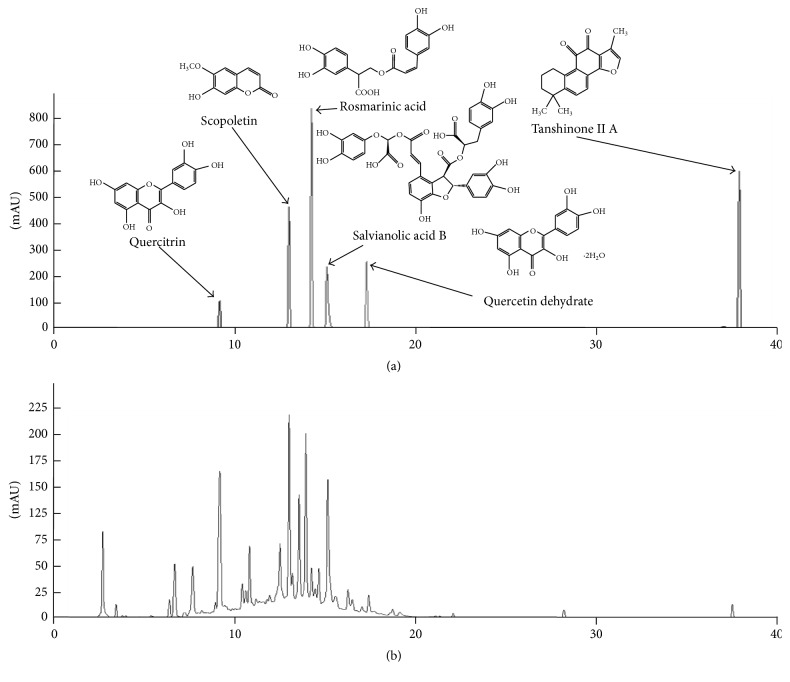
Chemical composition analysis based on high-performance liquid chromatography (HPLC). The water extract of CGXII and their standard compounds were subjected to HPLC. Chromatogram of reference compound mixtures (a) and CGXII (b). See Table 1.

The chemical constitutions of each individual herbal plant from the CGXII were evaluated using HPLC analysis. A total of six components, including scopoletin (in* A. iwayomogi*); quercitrin and quercetin dehydrate (in* A. xanthioides*); rosmarinic acid, salvianolic acid B, and tanshinone IIA (in* S. miltiorrhiza*), were observed in CGXII (Figures 1(a) and 1(b)). According to the results from the quantitative analysis, salvianolic acid, which is the most well-known reference compound, was most abundant in GGXII at 14.8 minutes of retention time (Figure 1(a)). The other compounds were detected at the following retention times: 12.7 min (scopoletin), 8.8 min (quercitrin), 16.8 min (quercetin dehydrate), 13.6 min (rosmarinic acid), and 37.2 min (tanshinone IIA) (Figure 1(b)). These chemical components ranged from 0.46 to 12.80 *μ*g/g (Table 1).

### 3.2. Effects on the Total Body and Organ Weights

DMN injection markedly lowered total body weights by 0.8-fold compared to that of the control group, and administration of CGXII did not normalize body weight. Absolute liver weight was not altered by DMN alone or DMN with CGXII treatment, but relative liver weight was notably increased in the DMN group compared with that of the normal group (approximately by 1.3-fold). Treatment with CGXII, particularly 50 mg/kg, significantly attenuated the elevation of relative liver weight (*p* < 0.05, Table 2). The DMN group also demonstrated considerable increases in absolute and relative spleen weights, compared with those of the normal group. Treatment with CGXII did not affect the weight changes produced by DMN. DDB (50 mg/kg) efficiently recovered the total body weights but not others.

### 3.3. Effects on the Liver Enzymes and Platelet Counts

DMN injection strikingly increased serum AST and ALT by approximately 9.6- and 18.3-fold compared with those of the normal group. Treatment with CGXII significantly attenuated the elevations of serum AST and ALT levels compared with those of the DMN group (*p* < 0.05 for 100 mg/kg in AST and ALT, Table 2). The platelet counts were markedly depleted by approximately 0.2-fold by DMN injection compared with those of the normal group, and CGXII did not affect them. DDB demonstrated a similar effect as CGXII platelet counts but showed the superior efficacy on both serum AST and ALT level.

### 3.4. Effects on Histopathological Findings

**Figure 2:**
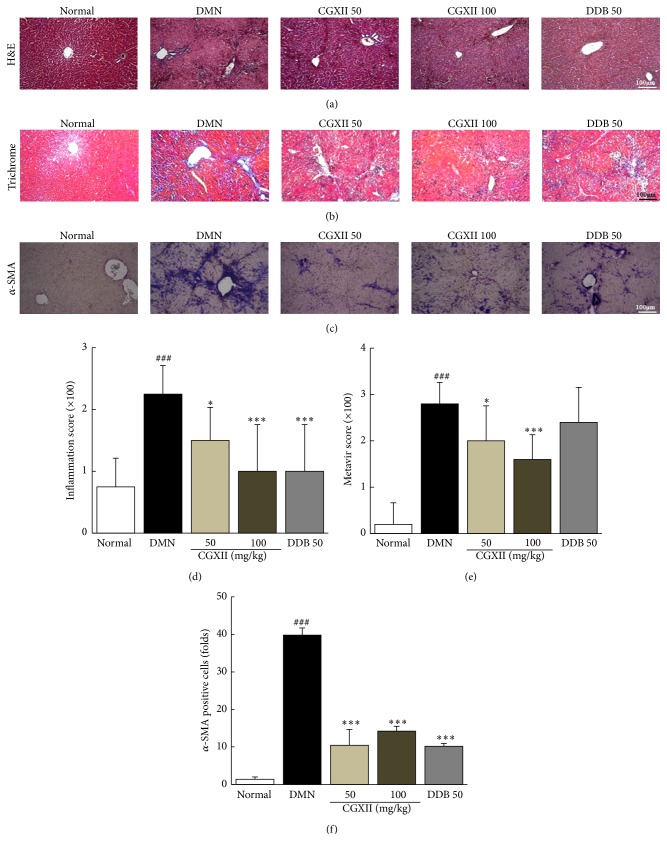
Histopathological analysis and immunohistochemical staining. Following DMN injection, rats were orally given distilled water, CGXII (50 mg and 100 mg/kg), or DDB (50 mg/kg) daily for 4 weeks. The liver tissues were examined using hematoxylin and eosin (a), Masson's trichrome (b), and immunohistochemistry for *α*-SMA (c); pathophysiologic examinations were performed under light microscopy (100x magnification). The inflammation scores (d), METAVIR scores (e), and the *α*-SMA positive cells (f) were analyzed. Data are expressed as the mean ± SD (*n* = 6). ^###^ *p* < 0.001, compared with the normal group; ^*∗*^ *p* < 0.05 and ^*∗∗∗*^ *p* < 0.001, compared with the DMN group.

The effects of CGXII on DMN injection-induced chronic hepatic injury were evaluated by histopathological examination of hepatic tissue using H&E staining. DMN injection resulted in a striking formation of bridging necrosis, inflammation, and wide infiltration of inflammatory cells around central veins, whereas CGXII significantly ameliorated this response (*p* < 0.05 for 50 and *p* < 0.001 for 100 mg/kg, Figures 2(a) and 2(d)). Masson's trichrome staining was performed to visualize collagen deposition in hepatic tissue. In the DMN group, substantial fibrotic change (blue staining) was shown, whereas both CGXII treatments significantly inhibited collagen accumulation in hepatic tissues (*p* < 0.05 for 50 and *p* < 0.001 for 100 mg/kg, Figures 2(b) and 2(e)). To investigate HSC activation, *α*-SMA level was analyzed by immunohistochemistry. The *α*-SMA signal (brown staining) was strongly enhanced by DMN injection; this effect was reduced by CGXII administration (*p* < 0.001 for 50 and 100 mg/kg, Figures 2(c) and 2(f)). DDB treatment also moderately attenuated these morphological alterations.

### 3.5. Effects on Hydroxyproline, Lipid Peroxidation, and Protein Carbonyl Content in Liver Tissues

**Figure 3 fig3:**
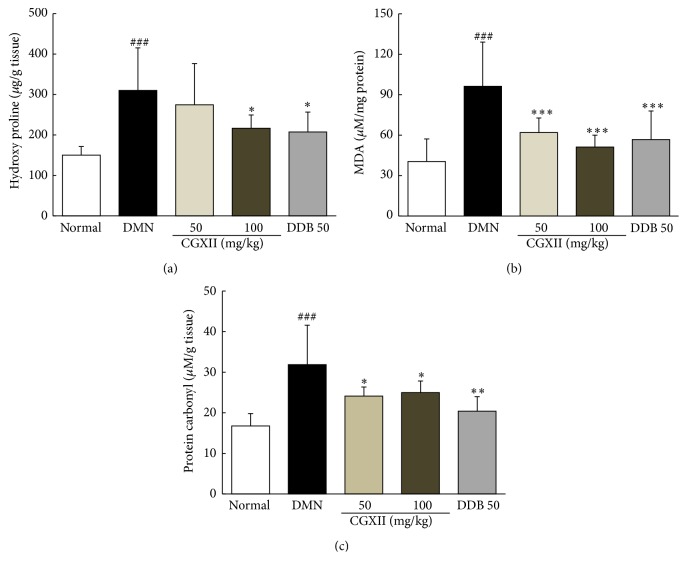
Contents of hydroxyproline, malondialdehyde, and protein carbonyl. Following DMN injection, rats were orally given distilled water, CGXII (50 mg and 100 mg/kg), or DDB (50 mg/kg) daily for 4 weeks. Hydroxyproline (a), malondialdehyde (MDA) (b), and protein carbonyl (c) content in the liver tissues. Data are expressed as the mean ± SD (*n* = 6). ^###^ *p* < 0.001, compared with the normal group; ^*∗*^ *p* < 0.05, ^*∗∗*^ *p* < 0.01, and ^*∗∗∗*^ *p* < 0.001, compared with the DMN group.

DMN injection dramatically increased the hepatic level of hydroxyproline (2.1-fold), MDA (2.4-fold), and protein carbonyl (1.9-fold) compared to those in the normal group. Administration of CGXII significantly reduced the hepatic level of hydroxyproline (*p* < 0.05 for 100 mg/kg), MDA (*p* < 0.001 for 50 and 100 mg/kg), and protein carbonyl (*p* < 0.05 for 50 and 100 mg/kg) compared with those of the DMN group (Figure 3). DDB showed an analogous effect on the levels of hydroxyproline, MDA, and protein carbonyl content (Figure 3).

### 3.6. Effects on the Protein Levels of Profibrogenic Cytokines and TIMP-1 in the Liver Tissues

**Figure 4:**
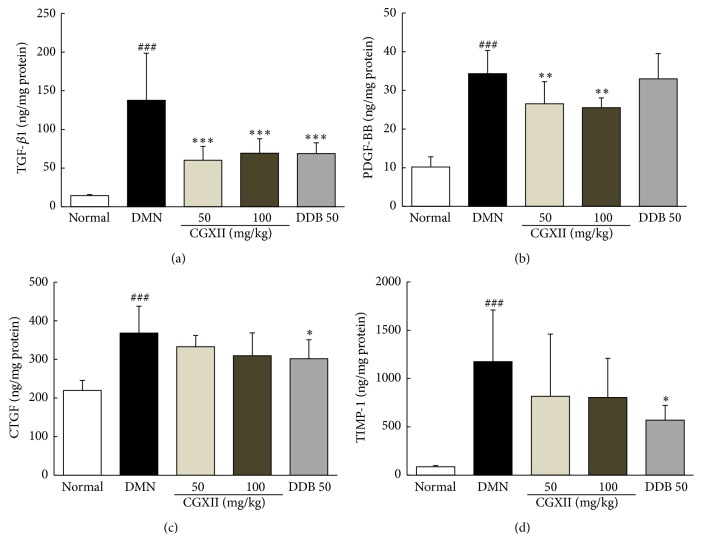
Determination of profibrogenic cytokines and TIMP-1. Following DMN injection, rats were orally given distilled water, CGXII (50 mg and 100 mg/kg), or DDB (50 mg/kg) daily for 4 weeks. Quantitative analysis of TGF-*β*1 (a), PDGF-BB (b), CTGF (c), and TIMP-1 (d) was performed in the liver tissues using ELISA kits. Data are expressed as the mean ± SD (*n* = 6). ^###^ *p* < 0.001, compared with the normal group; ^*∗*^ *p* < 0.05, ^*∗∗*^ *p* < 0.01, and ^*∗∗∗*^ *p* < 0.001, compared with the DMN group.

DMN injection substantially increased TGF-*β*1 by 10.0-fold compared with that of the normal group, whereas this abnormal elevation was significantly ameliorated by administration of CGXII (*p* < 0.001 for 50 and 100 mg/kg, Figure 4(a)). The DMN group showed remarkable increases in PDGF-BB of approximately 3.4-fold compared with that of the normal group, whereas CGXII significantly decreased the levels of PDGF-BB (*p* < 0.01 for 50 and 100 mg/kg, Figure 4(b)). Following the DMN injection both CTGF and TIMP-1 increased by approximately 1.7- and 13.6-fold, respectively, compared to the control group. Administration of CGXII did not significantly affect the levels of CTGF and TIMP-1. Administration with DDB also effectively improved the protein levels of TGF-*β*1, CTGF, and TIMP-1, but not PDGF-BB.

### 3.7. Effects on Fibrosis and Antifibrosis Related Gene Expression Analysis

**Figure 5:**
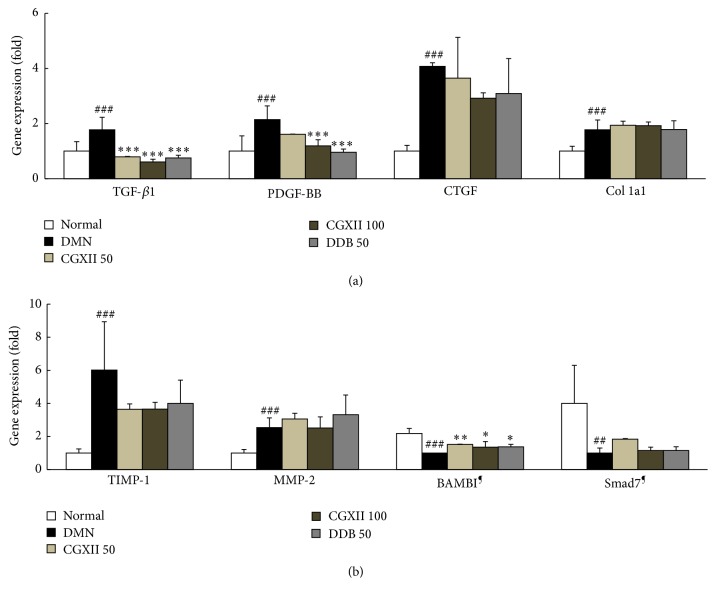
mRNA expression of hepatic fibrosis molecules. Real-time PCR was performed to determine the mRNA levels of TGB-*β*1, PDGF-BB, CTGF, Col 1a1 and TIMP-1, MMP-2 and BAMBI, and Smad7 in hepatic tissues. Expression was normalized as a ratio to *β*-actin. Data are expressed as the mean ± SD (*n* = 6). ^##^ *p* < 0.01 and ^###^ *p* < 0.001, compared with the normal group. ^*∗*^ *p* < 0.05, ^*∗∗*^ *p* < 0.01, and ^*∗∗∗*^ *p* < 0.001, compared with the DMN group. ^**¶**^Both BAMBI and Smad7 were normalized in the DMN group, and the others were expressed as fold changes, which were normalized in the normal group.

The gene expression levels of fibrogenic molecules including TGF-*β*1, PDGF-BB, CTGF, Col 1a1, and TIMP-1 were, respectively, higher in DMN group by 1.8-, 2.1-, 4.1-, and 1.8-fold compared with the levels of the normal group. The ECM turnover related molecules such as TIMP-1 and MMP-2 were markedly altered by DMN injection (6.0-fold higher in TIMP-1 and 2.5-fold higher in MMP-2 than that of the normal group). The antihepatofibrosis related molecules such as BAMBI and Smad7 showed remarkable downregulation by approximately 0.5- and 0.3-fold compared with those of the normal group. Administration of CGXII, however, significantly normalized TGF-*β*1, PDGF-BB, and BAMBI compared with those of the DMN group (*p* < 0.001 for 100 mg/kg in TGF-*β*1 and PDGF-BB, *p* < 0.01 for 50 mg/kg, and *p* < 0.05 for 100 mg/kg in BAMBI, Figures 5(a) and 5(b)). Administration with DDB also normalized the abnormal gene expression levels of TGF-*β*1, PDGF-BB, and BAMBI.

## 4. Discussion

Hepatofibrosis is a medical issue of great concern [27]. The social costs of treating hepatic fibrosis or cirrhosis have also steadily increased each year. The pathological mechanisms of hepatic fibrosis are unclear. Previously, many groups have made efforts to develop antihepatofibrosis agents, such as anti-TNF-*α* and UDCA [28, 29]; however no therapies have been approved. As a potential resource for therapies for hepatofibrosis, various herbal medicines have recently been suggested [30, 31].

Here, we aimed to determine the antifibrotic properties of CGXII in a chronic DMN-induced hepatofibrosis rat model. DMN has been widely used as an experimental model to study liver fibrosis [32, 33]. In our study, the DMN injection led to striking total body weight loss and increased the relative liver mass and spleen mass. The CGXII treatment resulted in a pattern of improvement with regard to these alterations, but no statistical significance was found except for relative liver mass (Table 2). One case of severe ascites (grade of 3) was observed in the DMN group but not in other groups (data not shown). The repeated DMN injection led to considerable hepatocyte destruction and inflammation, as evidenced by abnormal elevation of serum AST and ALT levels. These results were consistent with the histology findings, including inflamed cell infiltrations and necrotic cell bodies (Table 2 and Figures 2(a) and 2(d)). Additionally, total bilirubin in the serum level was elevated by 22-fold, and blood platelet counts were drastically depleted in the DMN group. The above alterations were significantly attenuated by CGXII (no statistically significant result in platelet count) in both histopathological findings and serum levels of hepatic enzymes analysis.

As we predicted, four-week DMN injections induced a moderate degree of hepatic fibrosis, with an average 2.8 METAVIR fibrosis scores as evidenced by Masson's trichrome staining (Figures 2(b) and 2(e)). This result is consistent with approximately 2-fold elevation in hydroxyproline content observed in the study (Figure 3(a)). Administration of CGXII significantly attenuated the changes in histopathology and the level of hepatic hydroxyproline induced by DMN. The CGXII treatment also improved the oxidative end product of lipid peroxidation determined by MDA, as well as the protein carbonyl content in hepatic tissues (Figures 3(b) and 3(c)). Oxidative stress is thought to participate in the pathological changes of hepatic fibrosis via continuous damage of hepatocytes [34, 35].

In the progression of hepatic fibrosis, HSCs play critical roles in producing ECM. TGF-*β*, PDGF-*β*, and CTGF, as profibrogenic cytokines, are known to be humoral factors that activate and stimulate the proliferation of HSCs, leading to excessive accumulation of ECM [36]. Accordingly, these cytokines are critical indicators of the pathogenesis of hepatic fibrosis [37]. DMN markedly activated HSCs as shown by immunohistochemistry staining of *α*-SMA, which is a potent marker of ECM, whereas CGXII opposed this action (Figures 2(c) and 2(f) [Fig fig3]). In our study, these three profibrogenic cytokines substantially increased both their protein and their mRNA expression levels in hepatic tissues in the DMN group; however these changes were significantly normalized, especially for TGF-*β*, in response to CGXII treatment (Figures 4(a)–4(c) and 5(a)). TGF-*β*, which leads to direct or indirect activation of HSCs, mainly induces the expression of PDGF-*β* and CTGF receptors [38]. Our results well demonstrated strikingly elevated protein levels of hepatic TGF-*β*, which plays a central role in hepatic fibrosis. PDGF-*β* is known to be a potent mitogen or activator of HSCs [39] and CTGF acts as a mediator of TGF-*β*-induced ECM formation in hepatic tissues in the progression of hepatic fibrosis [40]. TGF-*β* levels were greatly modulated by CGXII treatment, as compared with the response of the other two cytokines.

Additionally, activation of HSCs resulted in upregulation of collagen 1a1 mRNA in hepatic tissue. This event was not reversed by administration of CGXII (Figure 5(a)). BAMBI and Smad7 are well-known TGF-*β* inhibitors associated with a negative feedback response to TGF-*β* signals [41]. The gene expressions of BAMBI and Smad7 were notably downregulated in the DMN group, but this alteration was attenuated by BAMBI (Figure 5(b)). The accumulation of hepatic collagen is the consequence of an unbalance between excessive production and reduced degradation of ECM [42]. The ECMs are principally degraded by MMPs, while TIMPs are potent inhibitors of MMPs [43]. In the present study, we therefore explored the protein or mRNA expression level of MMP-2 and TIMP-1. DMN strikingly activated TIMP-1 (protein and gene expression) and upregulated the gene expression of MMP-2, whereas these changes were slightly attenuated by CGXII treatment, although not in a statistically significance manner (Figures 4(d) and 5(b)). The upregulated gene expression of MMP-2 was thought to be a compensatory response to excessive accumulation of ECM.

CGXII is originated from an antihepatofibrotic herbal formula, CGX, which was developed by the TOM theory for treating patients with chronic liver diseases. Additionally, CGX has been widely prescribed at the Oriental Hospital in republic of Korea since 2001, based on the clinical experience and many experimental data [18, 19, 44]. The CGX is composed of thirteen herbs including* A. iwayomogi*,* A. xanthioides*, and* S*.* miltiorrhiza*. We previously compared the antihepatofibrotic activities of thirteen herbs of CGX with respect to TGF-*β* inhibition using the HSC T-6 cell line [45, 46]. From those experimental data and practical theory of the traditional Korean pharmacology, we finally selected the abovementioned three herbal plants. The synergistic actions of three herbs of CGXII were proved comparing their individual abilities (data not shown). We have showed the six chemical compounds in CGXII; however we still lack knowledge regarding the active ingredient responsible

We have also reported the individual pharmaceutical activities of* A. iwayomogi *and* A. xanthioides *on hepatic injuries including hepatic fibrosis [47, 48].* S*.* miltiorrhiza *also affected hepatic fibrosis in an animal study [49]. This study, however, is the first to evaluate their antihepatofibrotic activity as a combination formula of* A. iwayomogi*,* A. xanthioides*, and* S*.* miltiorrhiza*. Generally, the traditional practices of herbal medicine include mixing multiple medicinal plants. The mixing of herbs has been believed to have higher activity and lower toxicity than individual herb administration, and this effect has been partially proved by a few experimental studies [50–52].

In this study, we used DDB as a positive control. The previous studies reported the antifibrotic effects and antioxidant effects of DDB animal models [20, 21]. The antihepatofibrotic properties of DDB were observed in our study, in which the activity of DDB (50 mg/kg) was roughly similar to CGXII (100 mg/kg).

## 5. Conclusions

Taken together, our findings demonstrated the antihepatofibrotic properties of CGXII in a DMN injection rat model. The underlying mechanisms responsible for the effects may involve the inactivation of HSCs through the regulation of fibrogenic cytokines, especially TGF-*β*.

## Figures and Tables

**Table 1:** The quantitative analysis of each component in CGXII.

Compounds	RT (min)	Mean ± SD (*μ*g/mg)
Quercitrin	8.8	16.16 ± 0.11
Scopoletin	12.7	10.97 ± 0.04
Rosmarinic acid	13.6	5.63 ± 0.04
Salvianolic acid B	14.8	12.8 ± 0.09
Quercetin dihydrate	16.8	0.71 ± 0.02
Tanshinone II A	37.2	0.46 ± 0.01

**Table 2:** Body and organ weights, serum biochemistries, and platelet counts.

Parameter	Normal	DMN	CGXII 50	CGXII 100	DDB 50
Body weight (g)	334.8 ± 18.0	259.8 ± 23.7^###^	270.3 ± 9.0	271.3 ± 19.4	286.2 ± 7.7^*∗*^
Liver weight (g)	10.2 ± 1.0	9.6 ± 1.7	9.1 ± 1.1	10.3 ± 0.9	11.1 ± 1.1
Relative liver weight (%)	3.0 ± 0.3	4.0 ± 0.3^##^	3.3 ± 0.4^*∗*^	3.3 ± 1.0	3.5 ± 0.4
Spleen weight (g)	0.8 ± 0.1	1.5 ± 0.2^###^	1.7 ± 0.1	1.7 ± 0.3	1.7 ± 0.2
Relative spleen weight (%)	0.2 ± 0.01	0.6 ± 0.09^###^	0.6 ± 0.05	0.7 ± 0.11	0.6 ± 0.07

AST (IU/dL)	173.3 ± 24.2	2180 ± 1983.2^##^	1061.6 ± 741.6	853.3 ± 359.5^*∗*^	481.6 ± 53.1^*∗∗*^
ALT (IU/dL)	38.3 ± 21.4	885 ± 515.9^###^	543.3 ± 212.2	478 ± 186.4^*∗*^	235 ± 51.3^*∗∗∗*^
Total bilirubin (mg/dL)	0.1 ± 0.0	2.2 ± 1.3^###^	1.1 ± 0.4^*∗∗*^	0.8 ± 0.1^*∗∗*^	0.6 ± 0.1^*∗∗∗*^
Platelet (k/*μ*L)	933.8 ± 41.2	222.8 ± 29.7^###^	251 ± 82.6	260.6 ± 143.2	290.3 ± 50.4

Following DMN injection, rats were orally given distilled water, CGXII (50 mg and 100 mg/kg), or DDB (50 mg/kg) daily for four weeks. Data are expressed as mean ± SD (*n* = 6). ^##^ *p* < 0.01 and ^###^ *p* < 0.001, compared with normal group; ^*∗*^ *p* < 0.05, ^*∗∗*^ *p* < 0.01, and ^*∗∗∗*^ *p* < 0.001, compared with DMN group.
